# A quality improvement approach to reducing hospital readmissions in patients with cancer and heart failure

**DOI:** 10.1186/s40959-019-0041-x

**Published:** 2019-06-10

**Authors:** Anecita Fadol, Joylynmae Estrella, Valerie Shelton, Maryam Zaghian, Diane Vanbenschop, Valerie Counts, Tito R. Mendoza, David Rubio, Patricia A. Johnston

**Affiliations:** 1 Department of Cardiology, The University of Texas MD Anderson Cancer Center, 1400 Holcombe Boulevard, Unit 0456, Houston, TX 77030-4009 USA; 2 Department of Nursing, The University of Texas MD Anderson Cancer Center, 1400 Holcombe Boulevard, Unit 0456, Houston, TX 77030-4009 USA; 3 Division of Nursing, The University of Texas MD Anderson Cancer Center, 1400 Holcombe Boulevard, Unit 0456, Houston, TX 77030-4009 USA; 4 Office of Performance Improvement, The University of Texas MD Anderson Cancer Center, 1400 Holcombe Boulevard, Unit 0456, Houston, TX 77030-4009 USA; 5 Information Services Division, The University of Texas MD Anderson Cancer Center, 1400 Holcombe Boulevard, Unit 0456, Houston, TX 77030-4009 USA; 6 Department of Symptoms Research, The University of Texas MD Anderson Cancer Center, 1400 Holcombe Boulevard, Unit 0456, Houston, TX 77030-4009 USA; 7 Department of General Internal Medicine, The University of Texas MD Anderson Cancer Center, 1400 Holcombe Boulevard, Unit 0456, Houston, TX 77030-4009 USA; 8 Cancer Network, The University of Texas MD Anderson Cancer Center, 1400 Holcombe Boulevard, Unit 0456, Houston, TX 77030-4009 USA

## Abstract

**Background:**

The management of patients with cancer and concurrent heart failure (HF) is challenging. The increased complexity of treatment and the occurrence of multiple overlapping symptoms may lead to frequent hospital admissions, which may result in cancer treatment delays, a diminished quality of life, and an increased financial burden for the patient’s family. To provide holistic care to oncology patients with HF, we implemented the Heart Success Program (HSP), a patient-centered, interprofessional collaborative practice, which decreased the 30-day hospital readmission rate for HF diagnosis from 40 to 27%. However, this rate remains higher than that reported for Medicare beneficiaries.

**Aim:**

To identify the factors contributing to frequent readmissions, the HSP committee participated in the institution’s Clinical Safety and Effectiveness and utilize quality improvement methodologies and tools to decrease hospital readmission for HF.

**Methods:**

The DMAIC (Define, Measure, Analyze, Improve and Control) method was used to guide this quality improvement. Areas considered as having high impact and requiring low effort to address were patient education barriers, lack of documentation clarity, and care provider knowledge gaps about the HSP. We implemented workflow changes, improved clarity with documentation of HF diagnosis, and increase provider knowledge about the HSP.

**Findings:**

After 6 months of implementing quality improvement techniques, the 30-day hospital readmission rate for HF patients fell by 23.43% (from 31.7% for the baseline period to 8.27%), exceeding the target project goal of 10%. Our quality improvement method may also be effective in improving the management of patients with cancer and other comorbid conditions.

## Introduction

The prevalence of cancer therapy-induced cardiotoxicity leading to heart failure (HF), one of the most dreaded complications of cancer therapy, is not well established because of the variable rates of cardiotoxicity among different types of anticancer agents. Anthracycline-induced cardiotoxicity, however, has been extensively studied owing to the long utilization of this drug class in the treatment of many malignancies. Data from the oncology literature indicates that more than 50% of patients treated with anthracyclines will exhibit some degree of cardiac dysfunction 10 to 20 years after chemotherapy, and 5% will develop overt HF [1]. The presence of HF in a patient with cancer limits the options for cancer therapy [2, 3]. In addition, managing patients with cancer and a concurrent HF diagnosis is challenging because of the increased complexity of treatment and multiple overlapping symptoms that result in frequent hospital admissions which increase the cost of care.

According to Medicare’s fee-for-service claims data, the 30-day hospital readmission rate for patients with HF was 22.3–24.8% [4–6], and Bell et al.’s [5] 2017 literature review on readmission rates among cancer patients found an all-cause hospital readmission rate within 30 days of 3–34%, with cardiopulmonary comorbidities consistently associated with higher rates of readmission. However, published data on 30-day hospital readmissions for patients with cancer and concurrent HF are lacking, most likely because the Centers for Medicare and Medicaid Services’ penalties for readmissions do not apply to cancer hospitals [7]. At our institution, a National Cancer Institute-designated cancer center, approximately 4% of patients discharged from the hospital have concurrent diagnoses of cancer and HF, and the 30-day hospital readmission rate for these patients is 40%, a rate much higher than Medicare patients [8]. These frequent readmissions can result in cancer treatment delays, negatively affecting a patient’s prognosis and quality of life and creating an increased financial burden.

To provide comprehensive holistic care to patients with cancer and HF, we implemented a patient-centered, interprofessional initiative, the Heart Success Program (HSP), in 2012. The HSP [9] reduced the 30-day hospital readmission rate from 40 to 27%. To further reduce the hospital readmission rate, the HSP committee then initiated a quality improvement process utilizing the DMAIC (Define, Measure, Analyze, Improve and Control) method [10] in 2017.

## Methodology

**Fig. 1:**
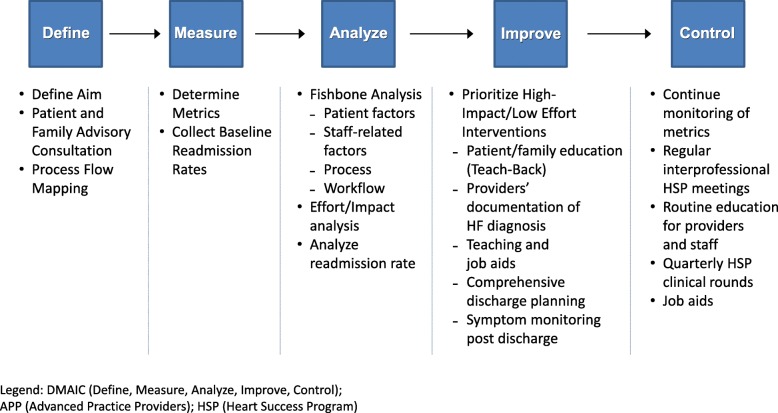
DMAIC Quality Improvement Methodology to Reduce Heart Failure Readmission

The five steps of the DMAIC method, a data-driven improvement cycle for optimizing and stabilizing processes and designs as applied to the HSP are discussed below, and summarized in Fig. 1.

### Define

The aim of our quality improvement project was to reduce the 30-day hospital readmission rate for patients with cancer and HF by 10% during a 6-month quality improvement initiative. To further define the problem, the HSP interprofessional team (from the departments of cardiology, oncology, nursing, rehabilitation services, information technology, and symptom management and research and the office of performance improvement) developed a baseline. For pilot implementation, the General Internal Medicine (GIM) unit for phase I clinical trials was selected because of its high volume of patients diagnosed with cancer therapy-induced cardiotoxicity.

### Measure

We reviewed the GIM database to identify patients discharged from the unit with a concurrent diagnosis of HF, defined by one of the following codes from the 10th revision of the International Classification of Diseases: 142.9 (cardiomyopathy), 150.9 (heart failure), 151.5 (myocardial degeneration), and 151.9 (left ventricular dysfunction). Then, we determined which of these patients were readmitted within 30 days with any of those HF-related codes. Of the 1083 patients discharged from the GIM unit from November 1, 2016 to March 31, 2017, the 5-month period prior to the launch of the quality improvement initiative, 120 (11.1%) had a concurrent HF diagnosis. Of those patients, 38 (31.7%) experienced a readmission within 30 days after the index hospital discharge for a HF diagnosis.

### Analyze

**Fig. 2:**
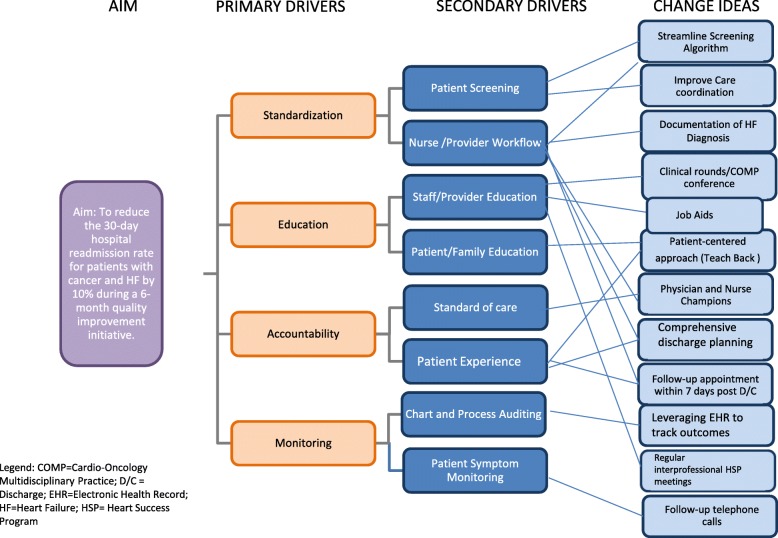
HSP Driver Diagram

We analyzed the root causes of 30-day hospital readmission through a brainstorming session that included input from three patients with cancer and a HF diagnosis— representatives from the institution’s Patient Family Advisory Council—who provided feedback on care processes from the patient perspective. Identified factors contributing to frequent hospital readmission were divided into categories, including patient, staff, and process and workflow factors, and incorporated into a fishbone diagram. Next, factors were categorized according to their importance (high, medium, or low) and the group’s ability to take action on them (in or out of control). Goals were translated to a set of key and secondary drivers (Fig. 2), directing us through brainstorming possible changes. The Impact Effort Analysis was used to prioritize the possible interventions suggested by the team. All possible interventions were rated on a scale from 0 to 10 according to their impact and the effort that would be required, and then they were graphically displayed in an implementation prioritization list. Among the interventions with high impact and low effort were those addressing patient education barriers, a lack of clarity of HF documentation, and care provider knowledge gaps about the HSP.

### Improve

To improve the 30-day hospital readmission rate, we disseminated the findings of the root-cause analysis to the GIM unit patient care team. Then, we addressed the high-impact, low-effort priorities identified during the Impact Effort Analysis. We revised the patient/family education workflow, improve documentation of HF diagnosis in the problem list, we created teaching and job aids to remind the patient care team of the changes to be implemented during the pilot, we implemented comprehensive discharge planning, and symptom monitoring post discharge.

### Control

To monitor the success of the interventions, a secure web application, Research Electronic Data Capture (REDCap™) was used to manage online surveys and databases [11]. Using a composite score, monthly audits were completed based on the core criteria (documentation of patient/family education; care plans and interventions; and discharge instructions) used in the HSP. In addition, the 30-day readmission rate was monitored and compared to baseline.

## Results

Upon completion of the 6-month quality-improvement program, 1256 patients were discharged from the GIM unit, 133 (10.6%) of whom had a concurrent diagnosis of cancer and HF. Of those, 40 (30.1%) were readmitted within 30 days of the index hospital discharge; however, only 11 (8.3%) readmissions were for HF-related reasons, while 29 (21.8%) readmissions were for reasons other than HF, including pneumonia, altered mental status, hypoxia, and fever. Thus, the 30-day hospital readmission rate for HF was reduced by 23.43% (from 31.7% for the 5-month period before the interventions to 8.3% for the intervention period), exceeding our target of a 10% reduction.

## Discussion

Our results show that the 30-day hospital readmission rate for patients with cancer and concurrent HF can be substantially reduced with the implementation of a quality improvement initiative. We attribute our success in reducing the readmission rate by 23.43% to our patient-centric focus, effective model of interprofessional collaboration, comprehensive discharge planning, and post-discharge support with follow-up phone calls to patients. Our process aligns with the Institute of Medicine call for a greater patient-centered focus; improved care coordination, with management of care transitions across continuum of care; and cost containment through the reduction of preventable healthcare use [5, 12]. Our results also agree with previous findings that interprofessional HF management teams can decrease readmission rates [13, 14].

Our next steps to expand the HSP throughout our institution will include educating patient unit staff regarding the newly improved workflow and the employment of a more efficient, patient-centered approach. Moreover, we will provide interprofessional education to physicians, advanced practice providers, and staff members in other disciplines, including nutrition and rehabilitation services, to alleviate challenges associated with timely enrollment of patients in the HSP consults, patient/caregiver education, and discharge planning. In addition, we will recruit HSP champions from specialty areas, including the pediatric unit, emergency center, pre- and post-anesthesia units, and the ambulatory departments to ease transitions of care.

To maximize the use of the electronic health record, we conducted initial brainstorming sessions with the Clinical Informatics team to address some of the barriers identified during the pilot, such as documentation of education, interdisciplinary care conferences, and discharge planning/instructions and the use of compliance reports for ease of monitoring and trending. Furthermore, as our organization invests in innovations in patient symptom reporting, we are actively participating in planning meetings to incorporate HF symptom monitoring and reporting.

To ensure sustainability in the implementation of the quality improvement process, metrics are tracked quarterly to ensure compliance with documentation of HF diagnosis, patient education, discharge instructions, hospital readmissions for heart failure diagnosis, and patient experience. In addition, regular interprofessional HSP meetings, routine educational offerings for providers and staff are conducted. Data from this continuous monitoring process identifies areas of deficiency that triggers intervention and maintains improved outcomes.

## Conclusion

Managing patients with cancer and concurrent HF is challenging and expensive, and health care costs are likely to increase as new, more advanced, and more expensive treatments are adopted as standards of care for both conditions resulting in increased survivorship. Future success in caring for patients with cancer and HF will depend upon the adoption of a broad perspective concerning patient needs, especially when patient needs challenge existing models of delivery. Our quality improvement initiative provides a model for engaging patients as partners in the shared goal of reducing the burden of HF in patients with cancer. Quality improvement efforts to reduce the substantial readmission rates among adults with cancer and HF might also be effective for patients with other conditions associated with high rates of hospital readmission, such as those with advanced cancer and multiple comorbid conditions.

## Abbreviations

CS & E: Clinical safety and effectiveness
DMAIC: Define, measure, analyze, improve, control
HF: Heart failure
HSP: Heart success program
